# Modelling the Relations of Rheological Characteristics with Composition of Plaster Mortar

**DOI:** 10.3390/ma15010371

**Published:** 2022-01-05

**Authors:** Khrystyna Moskalova, Tatiana Lyashenko, Aleksej Aniskin

**Affiliations:** 1 Department of Processes and Apparatuses in the Technology of Building Materials, Construction and Technological Institute, Odessa State Academy of Civil Engineering and Architecture, 4 Didrihsona St., 65029 Odesa, Ukraine; 2 Department of Information Technologies and Applied Mathematics, Educational and Scientific Institute of Business and Information Technologies, Odessa State Academy of Civil Engineering and Architecture, 4 Didrihsona St., 65029 Odesa, Ukraine; frabul16@gmail.com; 3 Department of Civil Engineering, University North, 104. Brigade 3, 42000 Varazdin, Croatia; aaniskin@unin.hr

**Keywords:** plaster mix, viscosity, thixotropy, design of experiment, Ostwald–de-Waele model, experimental-statistical model, effects of composition, computational experiment, correlation

## Abstract

The rheological properties of fresh plaster mortars, with varied contents of porous fillers and polymer admixtures, have been studied. The quantities of fine limestone and expanded perlite, and dosages of methyl hydroxy ethyl cellulose and ethylene vinyl acetate were varied in the experiment. Effective viscosity (at a shear rate from 0.045 to 5.705 s^−1^) and the thixotropy of the mixes were determined with rotational viscometer for 18 compositions (according to the design of the experiment). Each of the 18 viscosity curves were described with the Ostwald–de-Waele equation. The Experimental–Statistical models describing the dependencies of the parameters of the rheological model and of mix thixotropy on the composition factors were built on the obtained data. ES-models have allowed the individual and synergetic effects of mix components on the rheological characteristics to be evaluated. The expanded perlite powder can increase the viscosity by two times, probably due to its pozzolanic effect increasing the content of the CSH phase during cement hydration. The thixotropy can be increased by the quantity of limestone. The computational experiments with ES-models have made it possible for the information set, without a noticeable interrelation between rheological characteristics, to be stratified into subsets, in which such interrelations differ significantly.

## 1. Introduction

The developments in building material science, with advances in building physics, and new design methods have changed the attitude towards plaster materials. These changes have contributed to the expansion of requirements for the properties of building materials, and the compositions of modern plaster materials have become much more complicated.

The quality of the building materials is certainly dependent on the ingredients used in the building mix. The properties of the finished products are also dependent on the rheological behaviour of the fresh mixes [1]. In particular, the mixes for mechanized application should have some technological properties of hand-applied ones, and within a short time of mixing with water should ensure uniformity, have good pumpability and maintain working properties for a given time. And all the properties of fresh mixes, as well as of the finished products, are certainly defined by the composition of the mix.

To create high quality composite building materials, the multicomponent dispersed systems, more and more components are used, which could be multicomponent themselves. These could be mineral or polymer binder systems, mineral additives and complex chemical admixtures, poly-fractional fillers, hybrid fibres, etc. Also, it is quite difficult to predict the stability of a mix from basic technological properties. Some studies focused on the influence of composition, ingredient properties, and mixing procedure on the rheological properties of building mixes. As it has been noted in [2,3,4,5,6], the component of the cement mixtures plays a significant role, as the rheological properties are greatly related to the water-cementitious materials ratio, the presence of added cementitious materials, and chemical additives.

The relationship between viscosity and liquid structure has attracted considerable interest. There are several studies describing the influence of ingredients on rheological properties of cement composites [7]. The results in [8] demonstrated that addition of graphene oxide significantly modifies the rheological properties of the fresh cement pastes: leading to an acute increase in the yield stress, plastic viscosity, and the hysteresis loop area. Rostami et al. [9] have found that the superplasticizers were desorbed from nano silica surface and after that were adsorbed on the cement particles leading to decreasing the viscosity of cement suspension.

Most studies focus on the individual influence of each component on the mix. Typically, correlations between mixture content and rheological properties of the cementitious system are investigated [10]. Jiang et al. reported that the rheology of cement paste is correlated with the sort and amount of fillers, w/c ratio, and the content of superplasticizer. The authors [11] investigated rheological properties of cement mortar and blended mortar containing cement, fly ash, and blast furnace slag. The results have shown that in spite of the cement system and ternary system displaying similar yield stress, plastic viscosity, and static cohesion, the addition of additives had differing effects on each system. The incorporation of superplasticizers impacts more the yield–stress of the cement pastes and less the plastic viscosity [12]. Using the limestone and expanded perlite in building materials has environmental advantages and economic benefits, as noted in [13]. Therefore, it is necessary to investigate the influence from the use of lightweight aggregates in the stucco from the point to find its optimal dosages while maintaining the necessary technological qualities of the final material. The authors of [14] have noted that it is important to investigate the influence of the additives on limestone and cement pastes since the rheological processes are dependent on the chemical composition and reactivity of the various solids. Brumaud et al. [15] revealed the good correlation between measured yield stress and the adsorbed amount of cellulose ethers, and for hydroxypropoxy methoxy cellulose molecules tested in the study of cement pastes. Much better correlation between yield stress and the calculated desorption average force can be also seen in this work. The authors [16,17,18,19,20] also noted that the variation in the cellulose ethers’ dosage have a significant effect on the rheological properties including plastic viscosity and yield stress. In [17], the effect of hydroxyethyl methyl cellulose on various cement composites was studied and it was observed that the amount of the admixture used is the most important factor regardless of the type of binder. However, the authors focused their research on evaluating the influence of water rating admixture on the setting processes of mortars. The influence of cellulose ether on the rheological properties was not clearly assessed by them.

A more informative approach, which distinguishes our study from those mentioned above, would enable not only estimating rheological behaviour of the separate specific compositions, but evaluating the dependencies of rheological properties and the parameters of rheological equations on the compositions as well, getting the functions of them, has been put forward and used [21]. It was this approach that was used in the research described below.

Thus, the main value of the presented study lies precisely in obtaining the dependences of rheological characteristics, including thixotropy, on the composition of building mixtures.

The rheological properties, such as thixotropy and viscosity, are important physical characteristics. They can be used for a better understanding of the technological properties (flowability, stability, pumpability, etc.) of fresh paste for various building materials [22,23,24]. Thixotropy is especially important for the plaster mortars. When the mixtures are applied on a vertical surface, it is important to prevent their slipping and provide its original structure. Such thixotropic property is related to coagulation and dispersion of cement particles [6]. Even slight mixing changes the location of formed microflocculation units [25]. This characteristic of multicomponent cement paste has been poorly investigated.

Rheology can help in more specific characterization of cementitious materials and allows finding analytical and numerical explanations for specific flow properties. Moreover, this knowledge could be helpful in designing the cement-based compositions with various additives that modify the fresh mixes. Rheology is the fundamental basis for creating the dispersed systems and controlling their properties [26,27].

So, when developing high performance plaster mortars, it was important to study the rheological behavior of the technological mixes. The effects of the porous fillers and polymer admixtures (through which the quality of stucco could be controlled) on the rheological properties (viscosity, destruction rate, thixotropy) have been of special interest.

The aim of the paper is, firstly, to present the results of rheological measurements, with the corresponding rheological equations, for the number of plaster compositions, and secondly, to show the way the dependences of rheological characteristics of technological mixes on their compositions can be modelled on the basis of such data. Besides, it seems appropriate to present some results of using the obtained models for the analysis of mix components effects on the rheological properties and on the correlation between them.

## 2. Materials and Methods

### 2.1. Characteristics of Materials and Design of Experiment

In the study described below, the rheological characteristics (properties Y) of polymer–cement stucco mixes were studied. The components of the compositions are given in Table 1.

**Figure 1:**
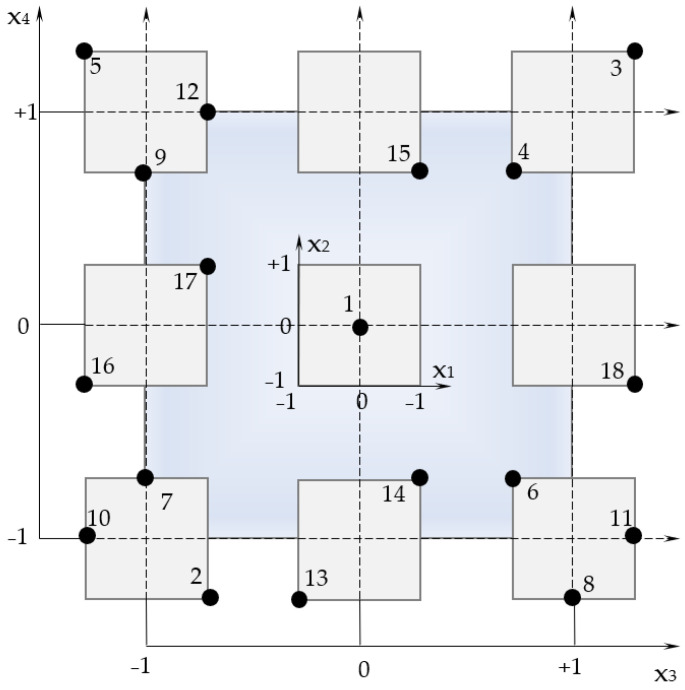
Points of the experiment design.

The quantities of 4 components (4 factors X) were varied in the experiment: ground limestone shell rock (marked X_1_), perlite sand (X_2_), cellulose methyl ether, 2-hydroxyethyl ether-Tylose MH60010 (X_3_), and copolymer powder of vinyl acetate and ethylene Vinnapas 5034N (X_4_). The contents of other components remained constant. The values of Y were determined for 18 compositions according to 18-points of 4-factor design of experiment [28]. The points of the design in coordinates of composition factors normalized to dimensionless −1 ≤ x_i_ ≤ +1 (instead of dimensional X_i_, X_i.min_ ≤ X_i_ ≤ X_i.max_) are shown in Figure 1 and given with corresponding compositions (natural factor levels X_i_) in Table 2.

This 3-level design of the 2nd order allows describing quantitatively, based on the data obtained, the individual and joint effects of composition factors on properties Y by 2nd order polynomial Experimental-Statistical (ES) model [29] of kind (1):

(1) $$Y\left(x\right)=b_{0}+\underset{i=0}{\sum }b_{i}x_{i}+\underset{i=1}{\sum }b_{ii}x_{i}^{2}+\underset{i<j}{\sum }b_{ij}x_{i}x_{j}$$

where: b represents the parameters (coefficients) to be estimated,

x—vector of normalized factors,
x_i_ = (X_i_ − X_0i_)/ΔX_i_, X_0i_ = (X_i.min_ + X_i.max_)/2, ΔX_i_ = (X_i.max_ − X_i.min_)/2
X_i_ = x_i_ · ΔX_i_ + X_0i._

Experimental-Statistical modelling (ESM) presents the methodology, the effective means for analysis and optimization of the structure, properties, parameters of operating and production of multicomponent building materials [16,29,30,31,32,33,34]. ESM [34] is the set of concepts, methods, and algorithms that connects the mathematical design of an experiment, regression analysis, and other means of applied statistics with a meaningful analysis of the obtained ES-models.

Experimental-Statistical modelling includes the following main steps [34]:

1. Predesign of the experiment, i.e., selection of the factors to be varied, limits of varying them, properties, the level of which should be determined, considering physico-chemical, technological, and other a priori knowledge.
2. Design of the experiment, with regard to the rational form of the polynomial model and possible existence of present experimental points and “forbidden” areas in the factor domain.
3. Building the regression model adequate for experimental data, with insignificant estimates eliminated.
4. Solving a variety of scientific and industrial problems, making decisions on the basis of individual ES-model (for separate criteria) and their complexity, extracting as much as possible scientific and industrial information from the models.

### 2.2. Testing Method

The experimental mix samples used for rheological measurements were prepared by an ordinary laboratory mixer. From the beginning of preparation all dry components were thoroughly mixed, after this, in the second stage, water was added in the dry mixtures. Water demand of the plaster mixture was determined by the diameter of the spread from the Hagermann cone by DIN 18,555 [35]. All samples should have 16–17 cm diameter of the spread. Cement pastes were mixed while controlling the consistency of the samples, adjusting the amount of water until the homogenous consistency was achieved. The total time of mixing the experimental mixes was nearly 5 min. After mixing, a portion of fresh sample (17 mL) was placed into a cylinder of rheometer for the rheological test.

The rotational rheometer used in this study was “Polimer” RPE-1M (manufacturer “Himpribor-1”, Tula, Russia) with coaxial cylinders measuring system according to DIN 53,018 [36]. The inner cylinder, i.e., the rotor, is run by a motor. Its speed is monitored for constant or programmed rotor speeds. At the same time, the second cylinder, i.e., the cup, remains at rest. The test was run at 8 values of shear rate γ′ (s^−1^), from the lowest γ′ = 0.045 to the highest 5.705 s^−1^ and back. The effective viscosity η (Pa·s) of each of the 18 mixes was measured sequentially 3 times by turning the rotor on and off. The average value of these measurements was taken as the measurement result. The duration of the measurement for each sample was 8 h. The temperature of the mortar was 19 ± 1 °C during the measurement and was kept at a pre-set level by the thermostatic chamber. The liquid in the annular gap is propelled to flow by the driven inner cylinder. It is possible to minimize errors during testing by making the gap size between cylinders smaller, linearizing the velocity gradient across radius of the cup and rotor [37]. So, we used type T2-B100 cylinders (manufacturer “Himpribor-1”, Tula, Russia). The inner diameter of the outer cylinder is 24 mm. The outer diameter of the inner cylinder is 17.479 mm. To define the annular gap size, we used the ratio of the radii, δ = 0.73. The DIN 53,018 standard [36] allows the use of devices, in which δ lies in the range 1 < δ < 1.10. The annular gap is of constant width: the test may be run with samples that contain particles of a particle size less than 1/3 of the gap size. The outcome of the resistance of the liquid being sheared between the stationary and rotating boundaries of the sensor system is a viscosity-related torque operating on the inner cylinder, counteracting the torque produced by the drive motor. Due to the torque applied by a spring twist, this torque detector is put between the drive motor and the shaft of the inner cylinder. The twist slope of the torque spring then serves as a direct measure of the viscosity of the sample.

### 2.3. Method of Measuring Theological Parameters

The viscosity curves of pseudoplastic fluids in certain range of shear rate can be described with the Ostwald–de-Waele Equation (2):

(2) $$\eta =K\cdot {\left(\gamma^{\prime }\right)}^{m}$$

The coefficient K is equal to the effective viscosity η_1_, Pa∙s, at shear rate γ′ = 1 s^−1^, and the exponent m < 0 characterizes the rate destruction of fluid structure during shear deformations-the higher |m|, the less stable the fluid structure during flow [37].

The parameters K and m in (2), which characterize a specific disperse system with a liquid phase (fixed composition), are referred to as the so-called “constants” of the physical model. However, the values of these parameters of rheological behaviour of the technological mix depend on its composition. This is reflected in the logarithmic form (3) of Equation (2):

(3) lnη (x)=lnK (x)+m(x)·ln γ’

**Figure 2:**
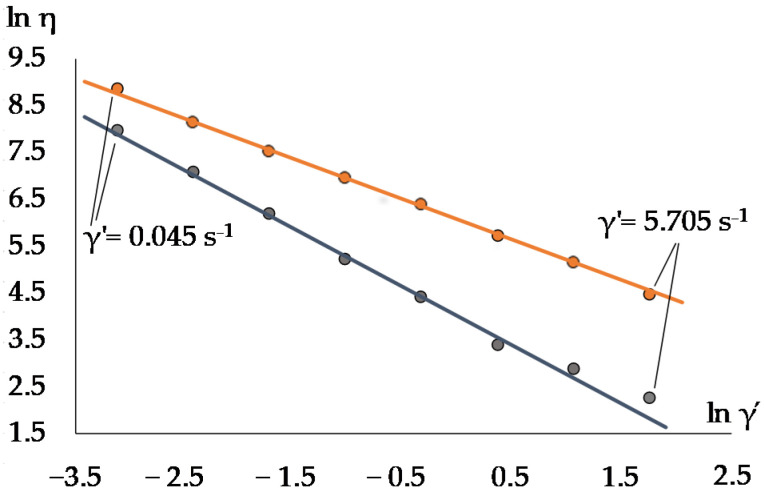
Logarithmic dependences of the viscosity on shear rate for compositions with minimum and maximum viscosity.

Figure 2 shows the results of plotting the viscosity curves for 2 compositions of 18, with the highest (418.8 Pa·s) and lowest (59.5 Pa·s) viscosity, respectively. The extreme points shown in Figure 2 correspond to viscosity at minimum γ’ = 0.045 s^−1^ and maximum speed γ’ = 5.705 s^−1^. For further analysis, we discarded these points in the calculations, since at the initial velocity the flow of the sample cement mixtures has not yet begun and the resulting deformations cannot be interpreted as an indication of yielding [38,39]. On the other hand, at high speeds, the behaviour of the sample is close to the viscosity of simple liquids with an extremely destroyed structure, where the critical deformation is the end of the linear elastic regime [40]. All rheological models obtained have the high coefficient of determination, R^2^ = 0.989 − 0.999.

**Figure 3:**
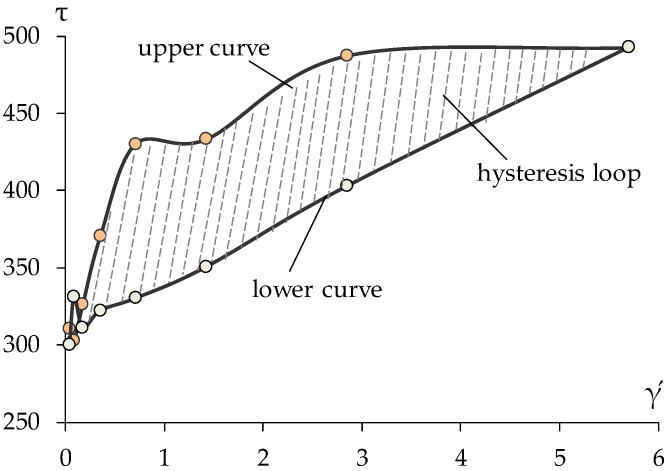
Thixotropy of the sample.

In order to calculate the thixotropy of investigated samples, we built flow curves obtained in the mode of uniform increase in the shear rate, the so-called “upper curves”, and “lower curves” obtained in the mode of reducing the shear rate. The area between upper and lower curves forming the so-called “hysteresis loop” is used as a measure of the thixotropic effect under consideration (Figure 3).

## 3. Results

### 3.1. Effects of Varied Components on Rheological Properties

The values of several rheological characteristics determined for 18 mortars under study are given in Table 3.

The values of K and |m| in Equation (2) for the 18 compositions made it possible to describe the dependences K (x) and m (x) by nonlinear ES-models of kind (1), in particular, the model (4) for K = η_1_ (with significant effects at experimental error of 4%), written in structured form.

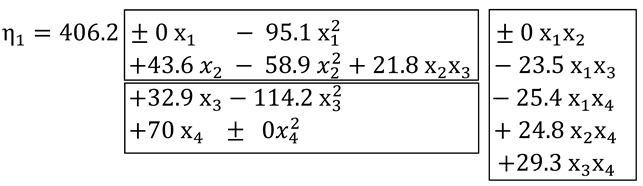
(4)

**Figure 4:**
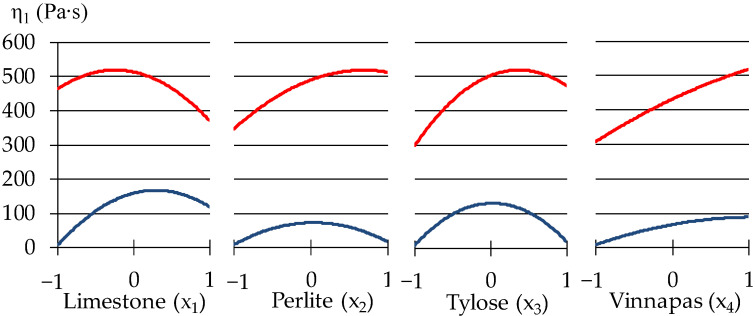
One-factor dependencies of η_1_ in zones of its minimum and maximum.

The free term b_0_ = 406.2 presents the level of η_1_ at medium values of all x, equal to zero, at the center of the experiment. The first block evaluates the effects of limestone and expanded perlite (so-called, effects of disperse phase) at central values of the factors from another group (quantity of polymer modifiers). The effects of those from another group at central values of the first group factors are presented in the second block. The third block evaluates the synergism or antagonism of the factors from two groups. The viscosity η_1_ described by Equation (4) has a minimum level η_1_._min_ = 18.24 Pa·s (at lower values of all 4 factors, x_1_ = x_2_ = x_3_ = x_4_ = −1) and maximum η_1.max_ = 511.45 Pa·s (at x_1_ = −0.19, x_2_ = 0.65, x_3_ = 0.4, x_4_ = 1). The influence of the additives on the viscosity may be analyzed from the graphs shown in Figure 4.

The results shown in Figure 4 indicated that the influence of Tylose on η_1_ is nonlinear: in concentration from 1 to 1.2 w.p., methyl hydroxyethyl cellulose increases the viscosity, but in higher amounts Tylose decreases the viscosity. Similar data of cellulose admixture in modified mortars were observed by other authors [41,42]. Such behaviour of Tylose can be attributed to the fact that cellulose might lubricate the particles of solid phase that lead to decreasing viscosity. However, the increase in viscosity can be associated with the ability of cellulose coming into contact with water, it begins an adsorption process, adhering to the periphery of the water molecules, imbibing and fixing part of the water in the mixture, thus increasing the viscosity of the material, as the authors of [43] note.

As Model (4) and Figure 4 show, the amount of Vinnapas (adhesion improving additive (x_4_) has the most substantial effect on the viscosity at the unit shear rate, when the mix is rather viscous. This indicates the possible strong bonds created by Vinnapas. It can be seen that the addition of expanded perlite powder to the mixes in quantities from 30 to 60 w.p. (by dry mix), increases the viscosity by two times in the zone of maximum. Similar results were obtained by the authors of [44], using 15–20% of expanded vermiculite in self-compacting mortars led to high viscosity at low rotational speed. The increase of viscosity can be ascribed to the pozzolanic effect of perlite, increasing the content of the CSH phase during cement hydration [45]. However, if the amount of other modifiers in mixes is low, this property of perlite weakens. The influence of limestone on the viscosity is less insignificant.

**Figure 5:**
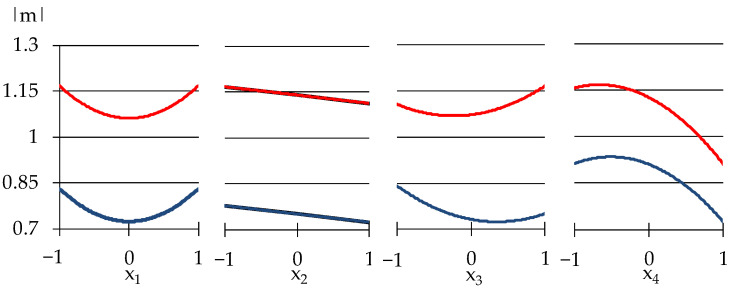
The influence of composition factors on the rate of structural destruction.

The results in Table 3 show that low-viscosity mixtures (η_1_ ≤ 157 Pa·s) at the same time could have a high rate of destruction (|m| > 1). This is a quite expected effect, which demonstrates that the created bonds are easily broken while mixing and at high values of |m| the mixtures lose their stability. In spite of this, some samples demonstrate stability of structure: No. 4, 15, and 12 have on average |m|_mid_ ≈ 0.87 while η_1mid_ ≈ 230 Pa·s. As can be seen in Figure 5, increasing the amount of Pelrite (x_2_) and Vinnapas (x_4_) from minimum to maximum will decrease the rate of structure destruction. The above analysis indicates that it is important to take the compatibility of the mixes’ components into consideration, since this can affect the conditions of processing the mortars and the stability of the structure.

**Figure 6:**
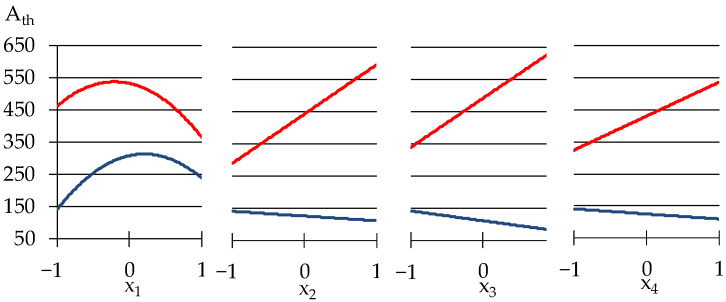
The influence of composition factors on the thixotropy of the mortars.

The thixotropy of investigated plaster mortars strongly depends on the contents of varied components, as can be seen in Figure 6.

Though the low thixotropy has a minor change with variation of perlite and additives dosages. This is indicated by the lower curve on the graphs for x_2_, x_3_, and x_4_. However, the content of limestone, x_1_, has a higher effect in the zone of minimum. When the minimum and medium amounts of limestone are added in mixes, the thixotropy doubles from 150 to 300 Wt/m^3^. With further increase of x_1,_ thixotropy decreases. The decrease of thixotropy can be observed in the maximum zone as well, A_th_ drops from 550 to 350 Wt/m^3^. Limestone is well known as an inert additive [46] which influences the flow properties of mixtures related with its different particle size. No relevant effects of limestone on rheological properties are confirmed by [47]: zeolite and silica fume have more effect on the rheological properties of fresh cement pastes than limestone, as it is indicated in his work. Not showing strong effect on viscosity, as mentioned above in our work, the limestone particles, however, can act as intercalated grains with strong nucleating properties and they enhance the thixotropy during the mixing process. In the study [48], also indicated the high thixotropy of the mortars with limestone and author associated this increase with a reduction in maximum packing density which, at a constant solid volume fraction in the suspension, leads to an increase in the contact interactions. Therefore, one of the efficient ways to create thixotropic mixes is to introduce hydration accelerating products, like limestone. The thixotropy increases with the contents of other modifiers only in the zone of its maximum, by 2–2.5 times. The effect described above can be related to the decrease of free water in mixes that leads to formation of the bigger microflocculation units complicating the movement.

### 3.2. Correlation between Rheological Characteristics

ES-models have also allowed the relations between rheological characteristics to be “measured” and analyzed. This could be useful, in particular, to answer the question: in what degree does mix thixotropy (A_th_) correlate to the rate of its structure destruction (|m|)?

As it has been pointed out in [49], “both parameters characterise the capability of pseudo-plastic fluid to resist the shear strain, which could either destroy or restore the structure” of the disperse system. Moreover, since the assessment of thixotropy requires the use of modern rheometers, and the value of |m| can be determined by two measurements on any viscometer with variable γ′, the analysis of correlation between A_th_ and |m| widens the possibilities for express controlling the quality of finishing mixes.

**Figure 7:**
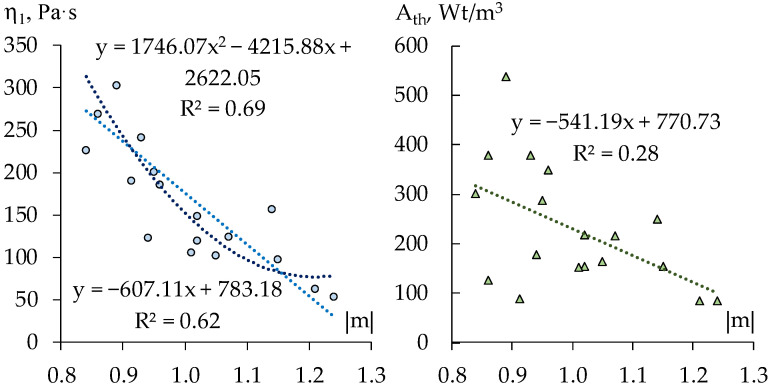
Scatter diagrams of viscosity at shear rate equal unity (K in Ostvald-de-Waele model), of thixotropy, and destruction rate of the 18 compositions (results of natural experiment).

The scatter diagrams in Figure 7 show some significant correlation between the rheological indices, judging by estimates of power model parameters obtained on experimental data for the 18 compositions; the coefficients of correlation *r* equal: r{K, |m|} = −0.78, r{A_th_, |m|} = −0.53. However, this should not be accepted formally, since these data could present essentially different structures of the mortar in various zones of formulation region (at this or that fixed dosages of mineral or organic components).

The information that would allow the existence of correlation and distinctions in relations between |m| and A_th_ in various formulation zones to be revealed and characterized can be obtained in computational experiments, in which the statistical trials (with Monte Carlo method [50,51]) are carried out [30,52]. The paired samples of any size (n) necessary for the analysis and for building possible prediction equations can be simulated with the help of the ES-models obtained. In each realization of the trial, the sufficiently great number of compositions (x) uniformly distributed in the whole formulation region under study or in its subregion are generated. For each x the generated normally distributed experimental error is added to the level of the property (Y) calculated by corresponding ES-model. By n pairs of these values the point estimate of coefficient r (or other measure of correlation) is obtained. Multiple realization of such trials makes it possible to get the distribution and interval estimate of r.

The procedure briefly described above was used in computational experiments, in various zones of the factor region, when the upper or lower levels of one or two factors were fixed (substituted in the models).

**Figure 8:**
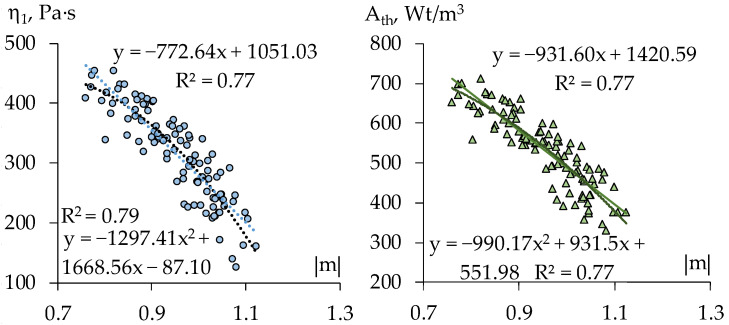
Scatter diagrams of the estimates of rheological characteristics at upper content of perlite and methylcellulose (x_2_ = x_3_ = +1) obtained in computational experiments with the help of ES-models (results of one trial).

**Figure 9:**
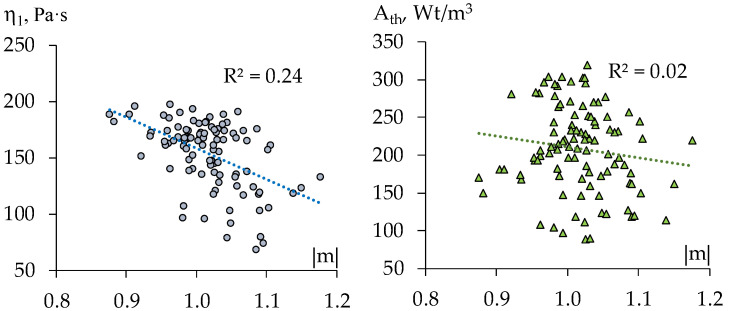
Scatter diagrams obtained in computational experiments at the lowest content of perlite and methylcellulose (x_2_ = x_3_ = –1).

Specifically, each diagram in Figure 8 and Figure 9 represents 100 pairs of values, (|m|, K) and (|m|, A_th_), for 100 generated compositions: uniformly distributed quantities of limestone aggregate (within the range 60 ≤ L ≤ 100 w.p., −1 ≤ x_1_ ≤ +1) and of dispersible polymer (within the range 1 ≤ V ≤ 2 w.p., −1 ≤ x_4_ ≤ +1), at maximal contents of perlite and methylcellulose (x_2_ = x_3_ = +1, Figure 8), and at the lowest values of these factors (x_2_ = x_3_ = −1, Figure 9).

As the diagrams show strong negative linear relation of the thixotropy with the rate of destruction revealed through varying the quantity of carbonate aggregate and the dosage of Vinnapas admixture at fixed high contents of perlite grains and of methylcellulose (average value of r, by several realization, r{A_th_, |m|} = −0.91) practically disappears with low content of these components (average r{A_th_, |m|} = −0.20, at right estimate +0.07). Thus, when one controls the structure of technological mix by varying the quantity of dispersible polymer in the matrix and the content of the aggregate the increase of |m| does imply the lowering of the thixotropy if the mixes contain “much” perlite and methylcellulose.

In most of the rest of simulated situations |m| defines A_th_ in the lesser degree, the certain other effects weaken the relation. The hypothesis of linear statistical relationship of K and |m| (evident in Figure 8) remains significant over most of factors subregions considered. In some cases, the non-linear hypothesis could be also admitted (as determination coefficients R^2^ in Figure 8 show).

## 4. Conclusions

The following conclusions based on the study presented above, can be drawn.

The viscosity curves (in the range of γ′ = 0.045 s^−1^ to 5.705 s^−1^ and back) obtained for each of the 18 plaster compositions in the designed experiment have been described with the Ostwald–de–Waele equation. The influence of the contents of limestone aggregate, perlite filler, Tylose, and Vinnapas in the compositions on the parameters of the power law model and on thixotropy has been evaluated with the help of ES-models.

The variation of the dosages of polymer additives and of the contents of porous perlite filler and limestone aggregate, within the studied intervals, changes the viscosity of the plaster mix at γ’ = 1 s^−1^ within the range from 18.24 to 511.45 Pa·s. The changes are related to the amount of physically bound water and the bonds created by the components of the mixture.

With the addition of limestone in quantities from 60 to 80 w.p. the thixotropy of mixes can be increased by two times. It is probably because of its nucleation properties and formation of the bonds between cement particles. Therefore, limestone benefits the structural build-up, in particular, thixotropy.

The addition of expanded perlite powder to the mixes in quantities from 30 to 60 w.p. (by dry mix), increases the viscosity two times. This effect can be attributed to the pozzolanic properties of perlite, which increases the content of the CSH phase, during the hydration of cement.

The computational experiments with ES-models have made it possible to stratify an information set (just a “heap of data”), without a noticeable interrelation between rheological characteristics, into subsets, in which such interrelations differ significantly. In particular, the strong negative correlation of the thixotropy with the rate of destruction has been revealed for compositions with high contents of perlite grains and of methylcellulose.

It is important that the joint models, combining rheological equations, such as Ostwald–de-Waele and others, with ES-models, such as (4), enable the great variety of engineering problems to be solved. These are:

- Estimating the viscosity of technological mix of any composition at any shear rate in the studied region;
- Inverse problems, of determining the shear rate that would provide the necessary viscosity of the mix, or of finding acceptable, optimal, and compromise compositions by rheological criteria together with operational properties of hardened composites.

## Figures and Tables

**Table 1:** Basic characteristics of used mixture components.

Mixture Components	Basic Characteristics
Portland cement	Additive-free cement produced by “Baltsem” M500 mark (PC I-500-N D0) (European quality certificate EN-197-1, CEM I 42.5 N). Specific surface is 300 m^2^/kg and fineness is 11.3%
Calcium hydroxide	Contention of CaO + MgO—73% by weight, water demand is 70%, bulk density is 0.5 kg/dm^3^
Limestone shell rock	Shell rock, with specific surface Ss.d. = 400 m^2^/kg, sifted through a sieve of 0.63 mm.
Quartz sand	Quartz sand from the Volnogorsk Mining and Metallurgical Combine. Density of quartz is 2.04 g/cm^3^, the particle size modulus is 1.1, the content of dust and clay particles is 0.3%, the clay content in the lumps is 0%, and the moisture content is 3.6%. The work used sand sifted through a sieve of 0.63.
Perlite sand	Perlite sand from the Beregovsky quarry of the Transcarpathian region. Expanded perlite fraction 0.16–1.05, grade in terms of bulk density 100, bulk density is 80 kg/m^3^, heat conductance at 25 ± 5 °C no more 0.052 Wt/m °C
Tylose MH60010	Water-retaining additive, methyl hydroxyethyl cellulose. Tylose is a water-soluble non-ionic cellulose ether, which is a derivative of the natural cellulose material.
Vinnapas RE5034 N	Adhesion improving additive, copolymer of vinyl chloride, ethylene, and vinyl laurate
Hostapur OSB	Air-entraining additive. Humidity—2%, sodium sulfate content of not more than 5.5%, potassium carbonate content of not more than 4%, bulk density—0.3 t/m^3^
Vinnapas 8031H	Water repellent, a triple copolymer of ethylene, vinyl laurate, and vinyl chloride. Bulk weight–450 ± 50 g/L, preferred particle size 0.3–9 microns, minimum glass transition temperature about 0 °C.

**Table 2:** Levels of composition factors in the experiment.

Number of Composition	Normalised Levels				Dosages (w.p. in 1000 w.p. of Dry Mix)			
	*x*_1_	*x*_2_	*x*_3_	*x*_4_	Limestone	Perlite	Tylose	Vinnapas
1	0	0	0	0	80	40	1.15	1.5
2	1	−1	−1	−1	100	30	1	1
3	1	1	1	1	100	50	1.3	2
4	−1	−1	1	1	60	30	1.3	2
5	−1	1	−1	1	60	50	1	2
6	−1	1	1	−1	60	50	1.3	1
7	0	1	−1	−1	80	50	1	1
8	0	−1	1	−1	80	30	1.3	1
9	0	−1	−1	1	80	30	1	2
10	−1	0	−1	−1	60	40	1	1
11	1	0	1	−1	100	40	1.3	1
12	1	0	−1	1	100	40	1	2
13	−1	−1	0	−1	60	30	1.15	1
14	1	1	0	−1	100	50	1.15	1
15	1	−1	0	1	100	30	1.15	2
16	−1	−1	−1	0	60	30	1	1.5
17	1	1	−1	0	100	50	1	1.5
18	1	−1	1	0	100	30	1.15	1.5

**Table 3:** The obtained values of rheological characteristics of the polymer–cementitious compositions.

No	Viscosity η_1_ (Pa·s)	The Error of Ostwald–de–Waele Model	Destruction Rate |m|	Thixotropy A_th_ (Wt/m^3^)
1	425	0.04	0.86	215
2	120	0.89	1.02	153
3	303	0.99	0.89	370
4	226	0.13	0.84	201
5	200.6	0.06	0.95	183
6	123.9	0.09	1.07	137
7	122.8	0.40	0.94	98
8	186.3	0.09	0.96	252
9	148.5	0.05	1.02	96
10	102.5	0.08	1.05	100
11	97.5	0.09	1.15	97
12	190.6	0.08	0.91	49
13	63.1	0.13	1.21	60
14	241.3	0.07	0.93	230
15	269.3	0.05	0.86	78
16	105.5	0.10	1.01	94
17	156.9	0.08	1.14	150
18	54.2	0.14	1.24	55
